# Physical Activity in Hospitalized Persons With Dementia: Feasibility and Validity of the Motionwatch 8

**DOI:** 10.1093/geroni/igaa057.2753

**Published:** 2020-12-16

**Authors:** Ashley Kuzmik, Barbara Resnick, Marie Boltz

**Affiliations:** 1 Pennsylvania State University, University Park, Pennsylvania, United States; 2 University of Maryland School of Nursing, Baltimore, Maryland, United States

## Abstract

Interventions to prevent functional decline in hospitalized persons with dementia (PWD) require objective measures of physical activity (PA). This secondary analysis described PA using MotionWatch 8 actigraphy and considered the feasibility and validity of the MotionWatch 8 in hospitalized PWD. In the first 320 PWD enrolled in the Fam-FFC study, 261 agreed to wear a MotionWatch for 24 hours within 48 hours of admission. Minutes were recorded in sedentary ( x̄ =1767.35, SD= 1327.43), low (x̄ = 202.52, SD=127.78), moderate (x̄ =7.93, SD=25.80 ), and vigorous activity ( x̄ = .85, SD=4.50). Controlling for age, gender, race and comorbidity, counts of activity were significantly associated with ADL function (t =4.3, p <.001). Sedentary (t =-3.9, p<.001), low (t =2.8, p =.006), and moderate (t =3.0, p =.003) activity, but not vigorous activity were significantly associated with ADL function. MotionWatch 8 appears feasible and valid when evaluating PA among hospitalized PWD.

